# Corrigendum: Identifying a set of influential spreaders in complex networks

**DOI:** 10.1038/srep31254

**Published:** 2016-08-25

**Authors:** Jian-Xiong Zhang, Duan-Bing Chen, Qiang Dong, Zhi-Dan Zhao

Scientific Reports 6: Article number: 27823; 10.1038/srep27823published online: 06 14 2016; updated: 08 25 2016

This Article contains errors in the Acknowledgements section.

“This work is partially supported by the National Natural Science Foundation of China under Grant Nos 61433014 and 61300018, by the National High Technology Research and Development Program under Grant No. 2015AA7115089, by the Fundamental Research Funds for the Central Universities under Grant Nos ZYGX2014Z002, ZYGX2015J152 and ZYGX2015J156 and by the Shanghai Research Institute of Publishing and Meida under Grant No. SAYB1402.”

should read:

“This work is partially supported by the National Natural Science Foundation of China under Grant Nos 61433014 and 61300018, by the Fundamental Research Funds for the Central Universities under Grant Nos ZYGX2014Z002, ZYGX2015J152 and ZYGX2015J156 and by the Shanghai Research Institute of Publishing and Meida under Grant No. SAYB1402.”

